# Minutes of the Ohio State Dental Society

**Published:** 1875-02

**Authors:** J. M. Porter


### Proceedings of Societies.

### MINUTES OF THE OHIO STATE DENTAL SOCIETY.

The ninth annual meeting of the Ohio State Dental Society was held in the Council Chamber at Columbus, Ohio, Dec. 3d, 4th and 5th, 1874. The meeting was called to order by Dr. H. A. Smith, President, and opened with prayer by the Rev. Hutchins. The minutes of the previous meeting were read, amended, and ordered filed.

The Executive Committee reported through the Chairman, Dr. Rehwinkle, and their report accepted. On motion of Dr. Rehwinkle, Thursday evening was set apart as a time for hearin the report of the Committee on Dental Patents, and for the consideration of this subject—motion carried.

The Committee on Membership, Publication and Ethics, asked further time—granted.

Dr. Rehwinkle asked what had been done concerning the publication of a list of all the dentists in the State, giving their professional status. Dr. Taft replied, that the list had not been published, because the list of names furnished by the Judiciary Committee was imperfect. The subject was discussed by Drs. Rehwinkle, J. Taft, Porter, Butler, R. G. Warner, Williams, J. H. Warner, C. R. Taft, Keely, and Whinery. Dr. Rehwinkle then moved that Dr. S. M. Porter be appointed Chairman of the Judiciary Committee, and given power to appoint an assistant in each Congressional District in the State, to assist him in completing this list of names. The motion was amended by Dr. Taft, giving this Committee the privilege to report at such times as they may think best, after consultation with the Publishing Committee. Motion adopted.

Dr. J. Taft moved that an appropriation not to exceed fifty dollars be made to defray the expense incurred by publishing this list of names. Motion adopted.

Drs. M. Wells, of Indianapolis, and J. R. Clayton, of Shelbyville, Indiana, were on motion of Dr. Keely, elected honorary members of this Society.

Dr. Rehwinkle moved that Dr. Emminger be appointed a Committee to convey the thanks of this Society to the Mayor and other City Officials of the City of Columbus, and invite them to attend the Sessions of this Socity. Drs. Rehwinkle and W. P. Horton were added to the Committee.

Dr. J. Taft moved that the following hours of meeting and adjourning be observed: Morning sessions, from 9 to 12 o'clock; afternoon sessions, from 2 to 5 o'clock; evening sessions, from 7½ to adjournment.

### FIRST DAY, AFTERNOON SESSION.

Society called to order by the President, Dr. H. A. Smith. Minutes of morning session read and approved.

The first subject for discussion: "Preservation of the Pulps of Teeth" was then taken up for discussion. This subject was classified as follows: "Systemic conditions, modifying treatment and influencing its results. The least injurious and yet efficient agents for covering and protecting dental pulp, and likewise promoting the formation of new (secondary) dentine. Causes of failure."

This subject was very fully discussed by Drs. Butler, Watt, Rehwinkle, J. Taft, Wells, J. H. Warner, Clayton, W. P. Horton, Porter and Jennings.

Dr. I. Williams, Chairman of Committee on Membership, reported the following persons as suitable for membership:

Frank A. Hunter, Cincinnati; F. W. Sage, Sandusky; A. L. Brown, Chillicothe; J. M. Whitney, Cleveland. On motion the Secretary was instructed to cast a ballot in their favor, and they were elected. On motion, adjourned.

### FIRST DAY, EVENING SESSION.

Society called to order by President, Dr. H. A. Smith. Minutes of afternoon session read and approved.

Dr. A. J. Douds, of Canton, was presented by the Committee on Membership, and elected.

The second subject: "Contour Fillings," was then taken up for discussion. This subject was classified as follows: "The conditions and circumstances demanding this kind of fillings. Failures and their causes."

This subject was freely discussed by Drs. Beauman, Keely Hunter, Williams, J. Taft, Butler, Rehwinkle, Clayton, French, Buffett, Whinery, Jennings, Watt, and W. P. Horton. On motion, adjourned.

### SECOND DAY, MORNING SESSION.

Society called to order by President Smith, and opened with prayer by Rev. Stedham.

On motion of Dr. Butler, the third subject was passed, and the fourth subject for discussion "Practical views and results of experience in the use of the various preparations of gold for filling teeth," be taken up.

This subject was classified as follows:

Cohesive gold—Is it as much used as formerly? Non-Cohesive gold—Reasons for preferring it to cohesive.

The discussion of this subject was opened by Dr. J. Taft, who was followed by Drs. Whitney, Rehwinkle, Butler, Beauman, Whinery and Horton.

President Smith announced the following gentlemen to conduct clinics, Thursday afternoon: Drs. Jennings, Keely, Beauman, Rehwinkle, Clayton, J. Taft and Emminger.

The third subject, "Status of Mechanical Dentistry," was taken up.

This subject was classified as follows:

Can the mechanical branch be rescued from its demoralized condition, and be raised to a higher and professional standing?

The best means for the accomplishment of this. Has the time arrived for the separation of the operative or surgical, from the mechanical department? Advantages and disadvantages of such separation, to each department.

This subject was opened by Dr. H. A. Smith, who read an essay on the subject. This essay included his retiring address as President. The subject was further discussed by Drs. Porter and Rehwinkle.

On motion of Dr. Taft, the fifth subject, "The best and most skilfull method of applying the Rubber Dam" be discussed in connection with the Clinics to be held this (Thursday) afternoon. Adjourned.

### SECOND DAY, AFTERNOON SESSION.

The first part of the afternoon was spent in demonstrating the Rubber Dam, and in examining instruments exhibited by Drs. Butler, Evans and J. H. Warner.

Prof. J. Taft demonstrated the use of a preparation for relieving the pain caused by excavating sensitive teeth, prepared by Drs. Cram and Melcher, of Chicago, called "Dental Pain Obtunder." Prof. Taft, by using this remedy, succeeded in excavating a large cavity without giving pain, that was very painful, when only cotton was applied, before this remedial agent was used in the cavity. Dr. Taft said he had used this preparation several months and found it all he could wish in making excavating a painless operation.

The Society was then called to order by President Smith, and on motion of Dr. Keely, the name of R. H. Boal, of Urbana, was favorably received, having been recommended by the Committee on Membership, and he was elected.

On motion of Dr. J. Taft, Dr. W. M. Herriott, of Indianapolis, was elected an honorary member of this Society. Dr. J. Taft then spoke of the importance of the use of the microscope, and exhibited several instruments to the Society. He was followed on the same subject by Dr. J. M. Whitney.

Dr. Jennings read a paper describing a very interesting case which resulted in a lady losing her tongue, as a result of suppuration.

This case was discussed by Drs. J. Taft, Jennings, Watt, Butler, and J. H. Warner. The paper of Dr. Jennings was referred to the Publishing Committee. Adjourned.

SECOND DAY, EVENING SESSION.

Society called to order by President Smith.

Dr. Jennings stated a case of malformation of teeth resulting in periostitis, and asked for advice on the subject. Dr. J. Taft presented his theory and mode of practice.

D. Rehwinkle, Chairman of the Committee on Dental Patents, submitted his report.

In addition, a lengthy letter was read from Messrs. Cox, Follett and Cochran, relative to the legal status of the Rubber Litigation. A statement was made that S. S. White, of Philadelphia, was ten thousand five hundred dollars out of pocket in his defence of the Rubber Suits, and that the test case, known as the "Smith case," which was decided against the dentists, in Portland, Maine, had been appealed to the Supreme Court of the United States, and is now waiting its turn on the calender for trial.

Dr. Rehwinkle stated that the question at issue between the owners of the Cummings' Patent and the dentists is, whether the rubber plate is legally a new article of manufacture in connection with the question of abandonment by Cummings of his invention.

Dr. W. M. Herriott stated a case of gross swindling on the part of the Rubber Co., against a dentist in Indiana.

On motion Drs. C. R. Taft and J. B. Beauman were appointed a Committee to audit the accounts of the Committee on Dental Patents, and report to-morrow morning.

The subject was further discussed by Drs. Horton, Rehwinkle, Brown and Smith.

The following resolution was offered by Dr. Rehwinkle, and adopted:

"*Whereas*, The Goodyear Dental Vulcanite Co. and Josiah Bacon, in circular dated Dec. 1st, 1874, and addressed to the Dental Profession, states among other things, that 'the Publisher of the Dental Cosmos,' has been engaged for several years past in advising in that magazine a course of litigation, which for some strange reason has been unfortunately followed by a great number of your profession."

This Association declares this statement to be contrary to the facts, and hereby testify that whatever Dr. S. S. White has done in connection with the rubber litigation, has been done by him in the interests, and by express solicitation of the Dental Profession, and we hereby tender him again our thanks. We furthermore request him to continue in the good work, and bring the case, now pending before the Supreme Court of the United States, to its final hearing, we pledging him material support.

On motion of Dr. Beauman the Committee on Dental Patents was re-appointed. This Committee consists of Drs. Keely, Williams, H. A. Smith, W. P. Horton and F. H. Rehwinkle. The thanks of the Society were, on motion, tendered this Committee for past labor. Dr. Keely then reported the results of his labor in collecting money for the Barnum fund.

Dr. Keely made an appeal to the members of the Society who had not paid anything in support of this "Barnum Testimonial Fund," to do so at once.

Dr. Taft moved that an assessment of ten dollars (in addition to the assessment made last year,) be made upon each member of the Society, for the purpose of defraying the expense incurred by the Committee on Dental Patents in defending suits brought by the Rubber Co. against dentists. Motion adopted.

On motion of Dr. Rehwinkle, the Treasurer was instructed to use his own judgment in collecting money assessed for the use of Committee on Dental Patents.

On motion of Dr. Whitney, a resolution of thanks was tendered Dr. Halderman for an invitation to visit the State Penitentiary to-morrow (Friday) morning. Adjourned.

THIRD DAY, MORNING SESSION.

Society called to order by President Smith.

The following bills were read, and ordered paid:

G. W. Keely, for Treasurer's Book, $3 50; Executive Committee, for printing circulars, $7 50; Secretary's bill for services and printing, $48 00; F. W. Sage, for reporting proceedings, $30 00; Janitor of Council Chamber, $10 00; Executive Committee, to printing, $2 00.

The Treasurer's report showed that he had received during the year $999 42. Paid out during the year $712 24. Balance on hand of $287 18.

The following officers were elected for the coming year:

President, Dr. C. R. Butler; Vice-Presidents, Drs. C. R. Taft and A. F. Emminger; Corresponding Secretary, Dr. Frank A. Hunter; Recording Secretary, Dr. J. N. Porter; Treasurer, Dr. G. W. Keely.

Drs. J. Taft and F. H. Rehwinkle were elected Members of the State Board of Dental Examiners for three years.

On motion, a vote of thanks was tendered the Press and Clergy of Columbus, for their kindness during the meeting.

Dr. C. R. Butler, the President elect, was conducted to the chair by Drs. Herriott and C. R. Taft. He returned thanks for the honor conferred; and expressed a hope that the relations between himself and all the members might be of the most pleasant character, etc.

Dr. Butler then announced the following appointments on the various Standing Committees for the coming year:

Executive Committee—F. H. Rehwinkle, J. H. Warner and D. R. Jennings.

Membership Committee—A. F. Eminger, A. L. Brown and J. C. Whinery.

Publishing Committee—J. M. Porter, J. Taft and J. B. Beauman.

Committee on Ethics—G. Watt, G. R. Warner and W. R. Lilly.

The Committee on Ethics reported two cases of violation of the Code of Ethics. These cases were referred back to the party who made the complaint, and to the Committee on Ethics. with instructions to report at next annual meeting.

The Secretary of State Board of Examiners reported that the Board had received on account of examinations during the past year, $225 00. Expense for past year, $230 25; leaving a deficit of $5 25, for which amount an order was given on Treasurer of State Society.

Dr. R. H. Boal offered the following resolution which was adopted:

"*Resolved*, that the Committee on Dental Patents be authorized to draw on the Treasurer for any funds in his possession, if in their judgment it is necessary to forward the work of the Commiteee."

Dr. W. P. Horton, Secretary of the State Examining Board, submitted the following as a report of their work for the past year:

TO THE OHIO STATE DENTAL SOCIETY.

*Gentlemen:*—Your Board of Examiners begs leave to make the followng report, viz:

In view of the modification of the law regulating the practice of dentistry, in this state, your board did not feel warranted in holding a semi-annual meeting, so that there has been but one meeting since the annual meeting in 1873.

This began its sessions on the first day of December 1874, at twelve o'clock, noon, at the Neil House, in the City of Columbus.

Six candidates presented themselves for examination. Of that number, three were found qualified, to whom certificates were granted, as follows: William S. Caris, Kent, Portage County; George W. Dills, Mansfield, Richland County; William T. T. Wallace, Cadwallader, Tuscarawas County.

Herrmann Wilde, from Germany, was examined at a special examination by Drs. J. Taft and H. A. Smith, and their favorable report confirmed by the board and a certificate granted.

There has been, since the law, under which we are acting, was amended in the Spring of 1873, a disposition on the part of some new beginners, and their friends, to evade its present provisions. What policy (if any) shall be adopted in view of this fact will be left for your wisdom to dictate.

Respectfully submitted,

W. P. Horton, Sec. J. Taft, Pres,

Drs. C. R. Taft and J. B. Beauman, the Committee appointed to audit the accounts of the Committee on Dental Patents, reported that they had carefully examined the accounts in detail, and all vouchers for expenditures, and find the same correct. Prof. J, Taft offered the following, which was adopted:

"In view of the fact that a few instances have come to the Board in which it would seem desirable to make a remission of certificate fee, therefore,

*Resolved*, That the Board of Examiners be authorized to exercise discretionary power in such cases, reporting them to the Society, except the name of the applicant.

Drs. F. A. Hunter and J. C. Whinery, Committee appointed to audit the accounts of the Treasurer, reported the same correct.

The subject of Dental Hygiene was discussed by Drs. Taft, Horton, Whinery and Rehwinkle, and the Society adjourned to meet on the first Tuesday in Dec., 1875.

J. M. Porter, Sec.

